# Direct contact with perivascular tumor cells enhances integrin αvβ3 signaling and migration of endothelial cells

**DOI:** 10.18632/oncotarget.9700

**Published:** 2016-05-30

**Authors:** Monica E. Burgett, Justin D. Lathia, Patrick Roth, Amy S. Nowacki, Deni S. Galileo, Elena Pugacheva, Ping Huang, Amit Vasanji, Meizhang Li, Tatiana Byzova, Tom Mikkelsen, Shideng Bao, Jeremy N. Rich, Michael Weller, Candece L. Gladson

**Affiliations:** ^1^ Department of Cancer Biology, Cleveland Clinic, Cleveland, OH, USA; ^2^ Department of Cellular and Molecular Medicine, Cleveland Clinic, Cleveland, OH, USA; ^3^ Department of Stem Cell Biology and Regenerative Medicine, Cleveland Clinic, Cleveland, OH, USA; ^4^ Department of Quantitative Health Sciences, Cleveland Clinic, Cleveland, OH, USA; ^5^ Department of Molecular Cardiology, Cleveland Clinic, Cleveland, OH, USA; ^6^ School of Biomedical Sciences, Kent State University, Kent, OH, USA; ^7^ Department of Neurology, Laboratory of Molecular Neuro-Oncology, University Hospital, Zurich, Switzerland; ^8^ Department of Biochemistry, West Virginia University, Morgantown, VA, USA; ^9^ Image IQ, Inc., Cleveland, OH, USA; ^10^ Department of Biological Sciences, University of Delaware and Helen F. Graham Cancer Center and Research Institute, Christiana Care Health System, Newark, DE, USA; ^11^ Department of Neurosurgery, Henry Ford Hospital, Detroit, MI, USA

**Keywords:** endothelial cells, glioblastoma, angiogenesis, integrin αvβ3, cancer stem cells

## Abstract

The secretion of soluble pro-angiogenic factors by tumor cells and stromal cells in the perivascular niche promotes the aggressive angiogenesis that is typical of glioblastoma (GBM). Here, we show that angiogenesis also can be promoted by a direct interaction between brain tumor cells, including tumor cells with cancer stem-like properties (CSCs), and endothelial cells (ECs). As shown *in vitro*, this direct interaction is mediated by binding of integrin αvβ3 expressed on ECs to the RGD-peptide in L1CAM expressed on CSCs. It promotes both EC network formation and enhances directed migration toward basic fibroblast growth factor. Activation of αvβ3 and bone marrow tyrosine kinase on chromosome X (BMX) is required for migration stimulated by direct binding but not for migration stimulated by soluble factors. RGD-peptide treatment of mice with established intracerebral GBM xenografts significantly reduced the percentage of Sox2-positive tumor cells and CSCs in close proximity to ECs, decreased integrin αvβ3 and BMX activation and p130CAS phosphorylation in the ECs, and reduced the vessel surface area. These results reveal a previously unrecognized aspect of the regulation of angiogenesis in GBM that can impact therapeutic anti-angiogenic targeting.

## INTRODUCTION

A highly angiogenic phenotype is a distinctive feature of glioblastoma (GBM), and is thought to contribute to the aggressive growth, invasive phenotype and post-therapy recurrence of these tumors [1–3]. In GBM and other cancers, pro-angiogenic signaling molecules, including basic fibroblast growth factor (bFGF) and vascular endothelial growth factor A (VEGF-A), are secreted by stromal cells and tumor cells. These factors promote endothelial (EC) activation, survival, protease secretion, sprouting and migration [2–5]. Our understanding of how the angiogenic process is regulated is not complete, however; for example, regulation of VEGF-induced sprouting in ECs by metabolic pathways was shown recently [6].

The migration of ECs towards paracrine-acting pro-angiogenic factors is mediated by integrins in cooperation with growth factors/growth factor receptors [7, reviewed in [8–10]]. Cell surface-expressed integrins recognize and are activated by ligands typically localized in the extracellular environment. This activation, in turn, results in activation of cytoplasmic kinases, such as Src and focal adhesion kinase (FAK), as well as adaptor molecules, such as p130CAS, resulting in reorganization of the actin cytoskeleton and generation of the mechanical force needed to pull the cell forward [7–12]. Several integrins recognize an RGD-peptide in their ligands. Cell adhesion receptors other than integrins, including L1CAM, also can promote cell migration [reviewed in [13]]. L1CAM binds to RGD-peptide-binding integrins, including integrin αvβ3 due to the presence of an RGD-peptide in its extracellular sixth Ig domain and this peptide appears to be necessary for the pro-migratory and pro-invasive effects of L1CAM in cancer cells [13–18].

In malignant tumors, a specialized perivascular microenvironment has been identified in which cancer stem-like cells (CSC) reside in very close proximity to ECs [19]. Use of integrin α6 as a marker of CSCs indicates that approximately 60% of integrin α6-positive cells reside within 5-μm of blood vessels in GBM [20]. It has been suggested that ECs maintain the CSC population, promoting the survival of the CSCs through secreted soluble factors (*e.g.*, nitric oxide) and laminin-α2 [20–23]. Conversely, the CSCs affect the ECs and promote angiogenesis through secretion of pro-angiogenic factors [4, 5]. There is some evidence that CSCs may interact directly with ECs; medulloblastoma CSCs bind to ECs plated on Matrigel^®^ and promote EC network formation [19] and melanoma cell contact with ECs in serum-containing media promotes expression of genes that regulate cancer cell migration and tumor progression [24]. Neither of these studies addressed the question of the mechanisms underlying the interactions or the identity of the cell type expressing the upregulated genes. Thus, the possibility that CSCs interact directly with ECs in the perivascular niche and affect EC behavior remains largely unexplored. We have examined direct interactions between primary brain ECs and CSCs derived from GBM or GBM cell lines and the subsequent signaling events using both *in vitro* approaches and an *in vivo* mouse model of GBM.

## RESULTS

### Direct contact of ECs and CSCs involving cell-cell adhesion mediated by integrin αvβ3 on ECs and L1CAM on CSCs

**Figure 1:**
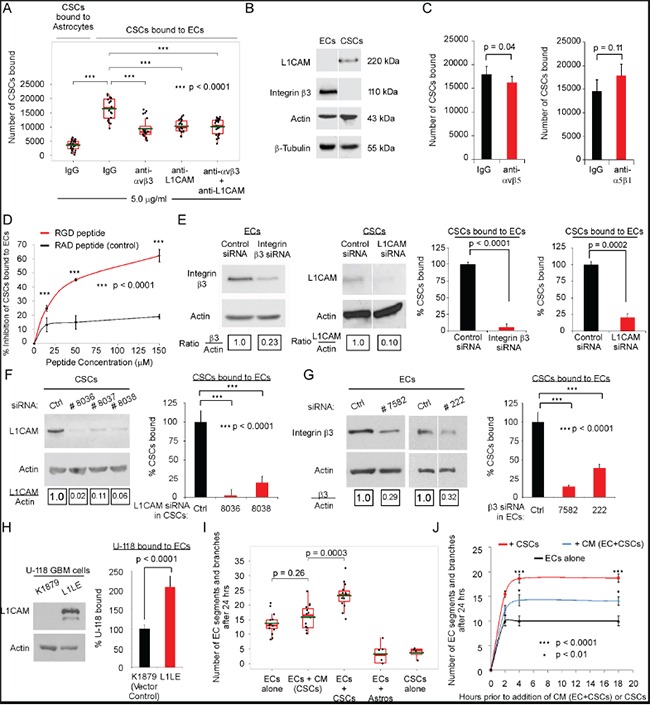
Integrin αvβ3 on ECs and L1CAM on CSCs mediate the direct contact of ECs and CSCs **A-H.** CSC adhesion measured by plating of GFP-CSCs-(08387) (3×10^4^) over unlabeled-ECs or astrocytes (5×10^4^/well) that had been seeded in serum-free adhesion assay buffer on plates coated with 10 μg/mL collagen. The GFP-CSCs-(08387) were incubated with the ECs or astrocytes (30 min), washed 3X with PBS and CSC adhesion to the EC or astrocyte monolayer measured by detection of GFP-CSC fluorescence using a fluorometer (485nm absorption, 535 nm emission) (replicates of five). (A-D) In some experiments, after overnight attachment, the seeded ECs or astrocytes were incubated with blocking antibody (A, C) or RGD peptide (D) or the corresponding controls for 30 min and the CSCs were incubated with blocking antibody (A, C) or RGD peptide (D) or the corresponding controls (30 min) prior to plating the CSCs over the EC or astrocyte monolayer. Expression of integrin β3 and L1CAM on adherent ECs and CSCs was determined by immunoblotting (B). (E-H) In some experiments, the ECs or CSCs were pretreated with pooled siRNA to β3 or L1CAM for 48h (E); or single target siRNA to L1CAM for72 h (F) or β3 for 72h (G); or adhesion of L1CAM overexpressing (L1LE) cells and U-118 MG cell vector control (K1879) to an EC monolayer was measured (H). **I–J.** Network/branch formation quantified as the number of segments/branches at 24 h after seeding of red-fluorescent ECs (20,000) alone or mixed with GFP-CSCs (20,000) or with astrocytes (20,000) onto Matrigel^®^ in complete NBM (I) or after seeding onto Matrigel^®^ in complete NBM 2, 4, or 18 h prior to the addition of CM (ECs+CSCs) or CSCs (J). Statistics: A, C, E-I, two-sided exact Wilcoxon rank-sum tests; D, two-way ANOVA; and J, repeated measure ANOVA. Graphs: A & I, Box and Whisker plots, C, E-H, data plotted as bar graphs, mean±SEM.

Using a cell-cell adhesion assay, we found that the CSCs readily adhere/bind to a confluent monolayer of ECs plated on collagen and that 4-fold more CSCs adhered/bound to ECs than to astrocytes (Figure 1A, SFigure 1A).

Integrin αvβ3, an RGD peptide-binding integrin that promotes EC adhesion, migration and survival [reviewed in [8, 9]], is upregulated on tumor-associated ECs in GBM biopsies [25]. The expression of the cell adhesion molecule L1CAM that contains an RGD-peptide is increased on CSCs from GBM [26]. Immunoblotting confirmed expression of the integrin β3 subunit on ECs and expression of L1CAM on CSCs (Figure 1B). An antibody that blocks integrin binding to the RGD peptide in L1CAM [14, 27] reduced CSC adhesion to ECs (Figure 1A). Pre-incubation of ECs with a neutralizing antibody to integrin αvβ3 or αvβ5 significantly reduced CSC adhesion to ECs (43% and 10%, respectively), but pre-incubation with a neutralizing antibody to α5β1 did not (Figure 1A&1C). As anti-integrin αvβ3 and anti-L1CAM in combination did not further inhibit CSC adhesion to ECs, integrin αvβ3 is most likely the major integrin involved in mediating this adhesion (Figure 1A). A cyclic-RGD-peptide significantly inhibited CSC adhesion to ECs in a concentration-dependent manner whereas a control RAD-peptide did not (Figure 1D). Downregulation of either the integrin β3 subunit on ECs or L1CAM on CSCs using pooled siRNA significantly inhibited CSC adhesion to ECs (Figure 1E). Similarly, downregulation of either the integrin β3 subunit on ECs or L1CAM on CSCs using two different single-target siRNAs for integrin β3 and two different single target-siRNAs for L1CAM significantly inhibited CSC adhesion to ECs (Figure 1F&1G). Moreover, overexpression of L1CAM in U-118 MG GBM cells (L1LE) [28] promoted the binding of GBM cells to ECs as compared to U-118 MG cells expressing the vector control (Figure 1H) and the expression of L1CAM on the 08387 CSCs promoted increased binding of CSCs to ECs as compared to the paired 08387 non-stem tumor cells (NSTCs) (SFigure 1B).

### CSCs from GBM promote network formation by brain ECs, activation of integrin αvβ3 and phenotypic changes in ECs

On co-seeding primary brain ECs with CSCs on Matrigel^®^, an interaction between ECs and CSCs could be seen at 2 h (SFigure 1C). The number of EC segments/branches (network formation) was higher on co-seeding of CSCs with ECs than when ECs were seeded in CSC-conditioned media (CM) (Figure 1I). This significantly higher number of segments/branches on co-seeding of CSCs with ECs than when ECs were seeded in CM (ECs+CSCs) was observed when the ECs were pre-seeded at 2, 4, or 18 h prior to the addition of CM (ECs+CSCs) or CSCs (Figure 1J). Thus, in subsequent experiments we differentiated the effects of direct contact to those of soluble factors by comparing the effects of co-seeding to the effects of CM.

For the subsequent experiments, we used laminin as the substrate as CSCs from GBM retain their stem cell phenotype when plated on laminin in neural basal media (NBM) [29] and integrin α6β1 mediates brain EC attachment to laminin [30].

**Figure 2:**
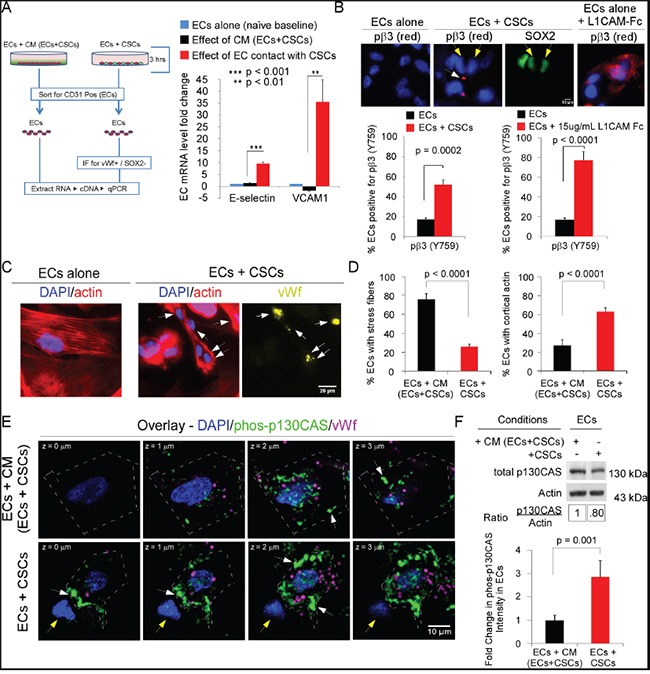
The direct interaction of ECs and CSCs activates ECs and promotes activation of integrin αvβ3 and p130CAS in ECs **A.** mRNA levels of genes marking EC activation were measured by qRT-PCR analysis of ECs seeded onto laminin in CM/EC+CSC (in growth-factor-free NBM) or mixed directly with CSCs-(08387) (growth-factor-free NBM) for 3 h (37°C, 5% CO_2_). ECs were then isolated by sorting for CD31. mRNA levels of genes after normalization to GAPDH are shown. **B-F.** Immunofluorescent analysis of markers of activation and signaling in ECs. ECs in CM/EC+CSC (growth factor-free NBM + 10 ng/ml bFGF) or mixed directly with CSCs (growth factor-free NBM + bFGF) were seeded onto laminin (3 h). Representative photomicrographs and quantification of phospho-β3 (Y759), a marker of integrin αvβ3 activation; white arrows denote phospho-β3 and yellow arrows denote CSCs (B); phalloidin-Alexa-594 (binds actin) and vWf (Alexa-647-yellow); arrows denote ECs with cortical actin or vWf expression (C-D); phosphorylated p130CAS (phos-p130CAS Y234; Alexa-488, green) and vWf (magenta); white arrows, green phos-p130CAS; and yellow arrows, CSCs (E). The intensity of green signal calculated using FIJI (ImageJ) based on the sum of the mean gray level (intensity) output of all pixels of the area within the enclosed cell traced along its outer edge, for each confocal section taken at 0.5 μm steps comprising the Z-stack. Signal intensity was normalized by dividing the sum of the mean gray level by the area of the cell. (F). Statistics: A, B, D and F, two-sided exact Wilcoxon rank-sum tests; data plotted as mean±SEM.

To determine whether the direct contact activates ECs, we analyzed mRNA levels of two markers of EC activation using qRT-PCR. The results indicated significantly higher E-selectin and VCAM-1 mRNA levels (9-fold and 34-fold, respectively) in ECs that were co-seeded with CSCs than in ECs seeded in CM obtained from co-seeded ECs and CSCs (CM/EC+CSC) at 3 h, indicating that EC activation was not induced by a secreted factor at this time point (Figure 2A).

Addition of recombinant L1CAM to ECs seeded alone resulted in activation of integrin αvβ3, as assessed by staining for phospho-β3 (pY759) [31, 32], confirming that integrin αvβ3 can be activated upon binding L1CAM (Figure 2B). The growth factor bFGF is upregulated in GBM; thus, to determine whether integrin αvβ3 on ECs is activated on binding L1CAM on CSCs, we co-seeded ECs and CSCs on laminin in the presence of bFGF (3 h) using Sox2 as a positive control for CSCs [33]. A significant increase in integrin β3 phosphorylation was observed when ECs were co-seeded with CSCs (Figure 2B).

**Figure 3:**
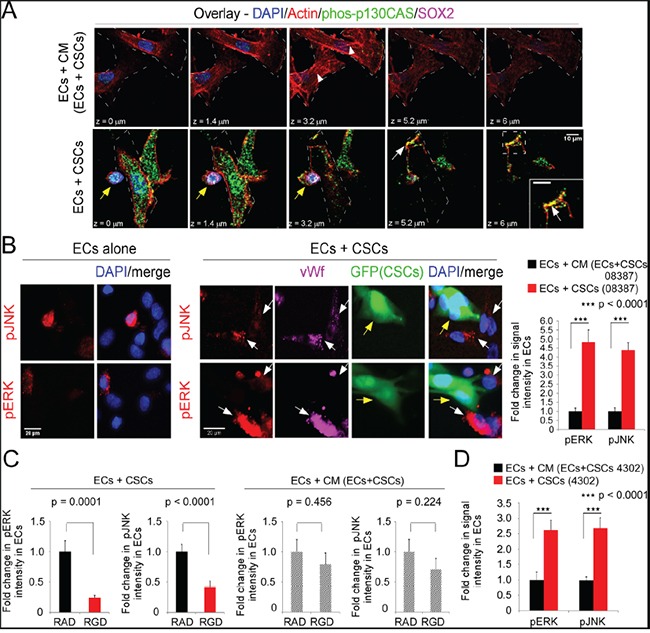
The direct interaction of ECs and CSCs activates ERK and JNK in ECs in an RGD-peptide-dependent manner ECs were seeded alone or with CSCs as in Figure 2B and markers detected by immunofluorecent labeling followed by confocal microscopy. **A.** Phosphorylated p130CAS (Y234) (Alexa-488, green) and actin (Alexa-594-Phalloidin, red). Sections comprising the Z-stack shown; white arrowheads-actin stress fibers; white arrows-co-localization (yellow) of phos-p130CAS and actin at membrane; and yellow arrows-CSCs. **B.** Phospho-ERK or phospho-JNK (Alexa-594, red) and vWf (Alexa-647, magenta). White arrows denote vWf-positive ECs expressing pERK or pJNK; and yellow arrows denote GFP-CSCs-(08387). **C.** RGD- or RAD-peptide (50 μM) were incubated with cells during seeding. **D.** ECs co-seeded with a second CSC isolate (4302) or in CM/EC+CSC; fluorescent intensity analyzed in 50 ECs and graphed as the fold-change. Statistics: B and C, two-sided student t-tests and D, two-sided Wilcoxon rank-sum test; B-D, Fold change in pERK or pJNK graphed as the mean±SEM.

Co-seeding of ECs with CSCs resulted in a phenotypic change in the ECs (Figure 2C). The number of ECs with stress fibers was significantly lower and the number with cortical actin was significantly higher as compared to ECs seeded in CM/EC+CSC (Figure 2C&2D). This suggested induction of a pro-migratory phenotype in the ECs on co-seeding with CSCs. As the phosphorylation of early downstream signaling effectors FAK and p130CAS is dynamic and depends on an organized actin cytoskeleton [reviewed in [8–10]], we quantitated the intensity of p130CAS phosphorylation (pY234) in confocal stacks of ECs. In ECs co-seeded with CSCs, phospho-p130CAS-(pY234) was nearly 3-fold higher than in ECs seeded in CM/EC+CSC (Figure 2E&2F); however, there was no significant difference in total p130CAS in ECs co-seeded with CSCs versus ECs seeded alone in CM/EC+CSC, based on cell harvest with Accutase, sorting for CD31, cell lysis and blotting for p130CAS (Figure 2F). FAK activity (pY397) was similarly increased in the ECs co-seeded with CSCs, as compared to ECs seeded in CM/EC+CSC (data not shown). Double-labeling for phosphorylated p130CAS and actin (Alexa-594-Phalloidin) followed by confocal microscopy showed focal co-localization at the membrane in ECs co-seeded with CSCs (Figure 3A). The downstream effectors ERK and JNK also can promote cell migration [34]. The activation of ERK and JNK was significantly higher in ECs co-seeded with CSCs from four different GBM tumors as compared to ECs seeded in CM/EC+CSC, and this increase was blocked by addition of RGD peptide (Figure 3B-3D; SFigure 2A). Collectively, these results suggest that the direct interaction between CSCs and ECs might represent a pro-angiogenic behavior of ECs as indicated by network formation and a transition in the ECs from a quiescent phenotype to an activated, migratory phenotype.

### CSCs increase the motility of ECs through direct contact as well as secreted factors

**Figure 4:**
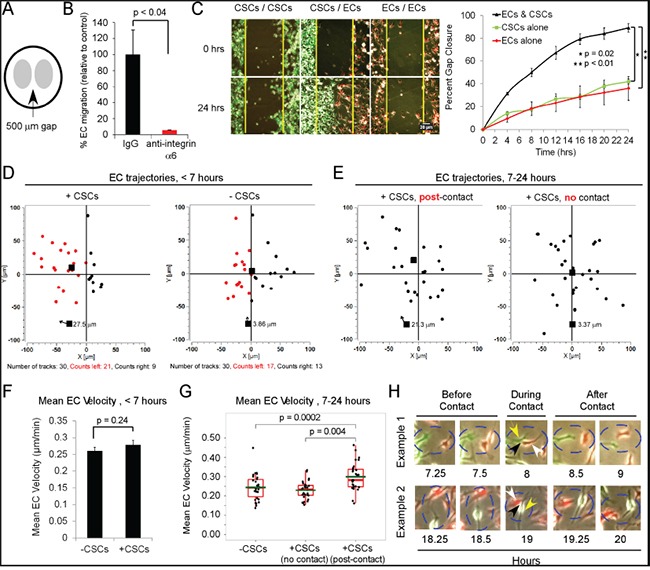
CSCs increase the directional 2D motility of ECs through both secreted factors and direct contact **A.** EC migration is mediated by an α6 integrin. Cell culture inserts were coated with 20μg/mL laminin, and red-fluorescent-ECs (30,000, EGM media) were plated on one side of the insert, and GFP-CSCs-(08387) (30,000, complete NBM) on the other side. At 18 h, all media were replaced with complete NBM; the insert removed, and live-imaging performed q 15 min (24 h, 37°C, 5% CO_2_). Migration was measured by counting each fluorophore in an automated fashion (customized software). **B.** In certain experiments, blocking anti-integrin α6 antibody or IgG as a control (5 μg/ml) was included in the complete NBM and nearly completely inhibited EC migration towards CSCs. **C.** Plot of gap closure. **D.** Paths of 30 ECs were manually traced in the first 7 h before EC-CSC contact and **E.** between 7-24 h when EC-CSC contact occurs using Manual Tracker (Image J). The final destination of each EC denoted on the graph, mean overall displacement-Euclidean distance (all 30 ECs) shown with the arrow. **F.** Mean EC velocity (displacement/time-μm/min) graphed as a bar graph of the mean±SEM**** or **G.** as Box and Whisker plots. **H.** Examples of ECs contacting CSCs in the gap area at various time points after 7 h; white arrows-ECs, yellow arrows-CSCs, and black arrowhead-point of EC-CSC contact. Statistics: C, F, and G, two-sided exact Wilcoxon rank-sum tests.

We investigated the effect of CSCs on EC migration using a 2D cell motility assay in which ECs and CSCs were seeded in NBM on laminin on either side of a 500-μm gap, and migration assessed over 24 h by live-imaging (Figure 4A). EC migration was almost completely inhibited by a neutralizing anti-integrin α6 antibody (Figure 4B). As compared to positioning of CSCs or ECs on both sides of the gap, the positioning of CSCs opposite ECs (on either side of the gap) significantly increased EC and CSC migration into the gap (10- and 14-fold, respectively; Figure 4C; SFigure 2B). Moreover, by 24 h the cells had filled the gap, whereas at this time point only 35% of the gap was filled when either CSCs or ECs were plated on both sides.

Live video imaging showed that contact between the ECs and CSCs first occurred after 7 h in this assay (Figure 4H); thus, the effects of potential CSC-secreted factors in the absence of direct contact could be assessed during the first 7 h. On tracing the paths of 30 ECs, we found that in the absence of CSCs (−CSCs), the ECs migrated randomly whereas when CSCs were present the majority of ECs displayed a preferential migration in the direction of the CSCs (negative X axis) (Figure 4D; SFigure 2C). During the first 7 h, there was no significant difference in the velocity of the ECs when plated opposite CSCs or ECs (Figure 4F). Thus, potentially, CSC-secreted factors promote the directional motility of ECs but do not alter the velocity.

We then assessed the effects of direct contact of CSCs with ECs on EC migration by tracing trajectories during the 7-24 h time frame. We compared the trajectories of 30 ECs that had been observed to make contact with CSCs during the 7-24 h time frame with the trajectories of 30 ECs that never contacted CSCs but were plated opposite of CSCs, over the same time frame. We found that the ECs that had been observed to contact CSCs exhibited a significantly greater displacement and velocity (21.3 μm and 0.3 μm/min, respectively), and exhibited directional migration, compared to the ECs that had never contacted CSCs (3.4 μm and 0.23 μm/min, respectively) (Figure 4E&4G; SFigure 2D). ECs that were seeded in the absence of CSCs continued to display random migration (SFigure 2E).

**Figure 5:**
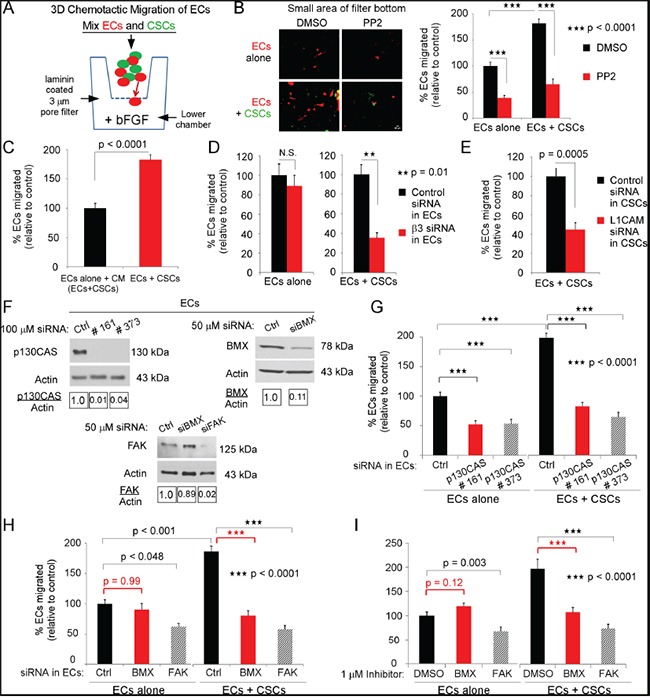
The direct interaction of ECs and CSCs promotes EC migration towards bFGF **A.** Diagrammatic representation of experimental design. Filters (3-μm pore) were coated on both sides with 20 μg/mL laminin. Red-fluorescent-ECs (3×10^4^) were seeded alone or mixed with GFP-CSCs-(08387) (3×10^4^) and co-seeded in growth factor-free NBM with 1% BSA on top of the filter. Growth factor-free NBM with 10 ng/ml bFGF was placed in the bottom chamber, and the cells allowed to migrate (37°C, 5% CO_2_). At 6 h, cells were removed from the upper filter surface and cells on the lower filter surface were washed, fixed, photographed and quantified. **B.** The Src inhibitor PP2 (1 μM) was incubated with ECs (20 min), followed by washing, and EC seeding alone or co-seeding with CSCs. **C.** ECs were seeded in CM/EC+CSC (described in Figure 2B), or co-seeded with CSCs. **D, E.** ECs were treated with integrin β3 specific siRNA, L1CAM specific siRNA or with control siRNA for 48 h, washed and seeded alone or with GFP-CSCs. **F-H.** Downregulation by the indicated siRNA or control siRNA (48 h FAK and BMX, 72 h p130CAS) in ECs was confirmed by immunoblotting (F), then ECs were treated with p130CAS, FAK or BMX specific siRNA or with control siRNA, washed and seeded alone or with GFP-CSCs. **I.** ECs were treated with 1μM of the FAK or BMX inhibitor or DMSO (control), washed and seeded alone or with GFP-CSCs. Statistics: B-E, G-I, two-sided exact Wilcoxon rank-sum tests, and data graphed as the mean±SEM.

To assess the effect of EC-CSC contact on EC migration towards a chemo-attractant, we used a Transwell^®^ assay with laminin-coated 3-μm pore filters to mimic the tight spaces in the brain extracellular matrix [35] (Figure 5A). Red-fluorescent-ECs were mixed, co-seeded with GFP-CSCs in the upper chamber and allowed to migrate for 6 h towards bFGF in NBM in the bottom chamber. Co-seeding of ECs with CSCs in the top chamber significantly increased EC migration as compared to migration of ECs seeded alone in the top chamber (Figure 5B). The increase in EC migration when co-seeded with CSCs was not replicated by seeding ECs in CM/EC+CSC, indicating the increase in EC migration was not due to a secreted factor(s) (Figure 5C).

Src phosphorylates the substrate domain of p130CAS, which is necessary for p130CAS activation [reviewed in [8–10]]. Preincubation of ECs with the Src inhibitor 4-amino-5-(4-chlorophenyl)-7-(dimethylethyl)pyrazolo[3,4-α]pyrimidine (PP2) for 20 min followed by washing and seeding significantly inhibited EC migration towards bFGF whether the treated ECs were co-seeded with CSCs or seeded alone (Figure 5B). Downregulation of β3 in ECs resulted in a significant decrease in migration of ECs co-seeded with CSCs (Figure 5D), but had no significant effect on the migration of ECs that were seeded alone (Figure 5D). This was consistent with our observation that integrin αvβ3 is not mediating adhesion or migration of ECs on laminin. Downregulation of L1CAM on CSCs also resulted in a significant reduction in migration of ECs co-seeded with CSCs (Figure 5E). Collectively, these data suggest that the interaction of CSCs with ECs activates signaling effectors in ECs thereby promoting migration towards bFGF that is not mediated by binding of integrin αvβ3 to the laminin substrate.

### Bone marrow tyrosine kinase on chromosome X (BMX) as well as FAK and p130CAS are required for enhanced migration of ECs towards bFGF when co-seeded with CSCs

To determine whether p130CAS was necessary for the enhanced migration of ECs when co-seeded with CSCs, we downregulated p130CAS in the ECs with two different single target siRNAs or pooled p130CAS siRNA (Figure 5F, SFigure 3A). This resulted in a significant inhibition of EC migration towards bFGF when co-seeded with CSCs or when seeded in CM/EC+CSC (Figure 5G, SFig 3B). Both FAK and BMX can phosphorylate p130CAS, although at different sites, and contribute to p130CAS activation [8–10, 36]. When FAK was downregulated in the ECs, we found a significant inhibition of EC migration whether the ECs were co-seeded with CSCs or seeded in CM/EC+CSC (Figure 5F&5H). In contrast, when BMX was downregulated in the ECs we found a significant inhibition of EC migration when the ECs were co-seeded with CSCs, but not when they were seeded alone in CM/EC+CSC (Figure 5F&5H). Supporting these findings, the use of a FAK or a BMX inhibitor (1μM) showed highly similar effects on EC migration as compared to downregulation of FAK or BMX expression, respectively (Figure 5I). This indicates a differential requirement for BMX for the increased chemotactic migration of ECs when they are co-seeded with CSCs.

**Figure 6:**
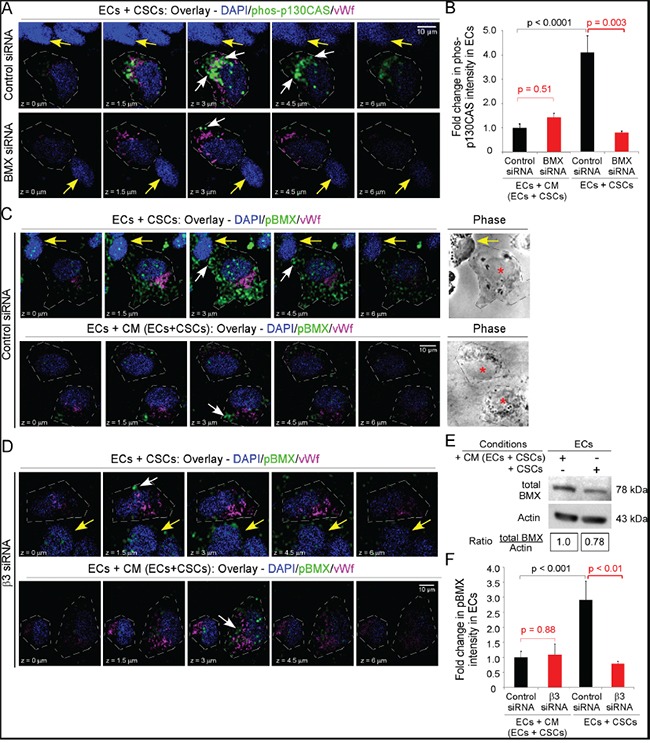
Integrin αvβ3 is necessary for BMX activation and BMX is necessary for p130CAS activation in ECs contacting CSCs ECs were seeded alone or with CSCs-(08387) as in Figure 2B. **A-B.** ECs were treated with BMX siRNA or control siRNA (48 h), followed by seeding as above and immunofluorescence for phos-p130CAS (Y234) (Alexa-488, green) and vWf (Alexa-647, magenta, marker of ECs). Confocal sections (0.5-μm apart) comprising the Z-stack are shown. White arrows denote phos-p130CAS and yellow arrows denote CSCs (vWf-negative) (A). The intensity of phos-p130CAS was determined in 15 representative cells as in Figure 2E&2F and graphed as the fold-change. (B). **C-F.** ECs were treated with control siRNA (C) or integrin β3 siRNA (D) for 48 h, followed by seeding as above and immunofluorescence detection of phospho-BMX (pBMX) (Alexa-488 green) and vWf (Alexa-647, magenta, EC marker). Confocal sections (0.5-μm apart) comprising the Z-stack are shown. White arrows denote pBMX, yellow arrows denote CSCs (vWf-negative) and red asterisks denote ECs in phase contrast images (C,D). The intensity for pBMX was determined in 15 representative cells as in Figure 2E&2F and graphed as the fold-change. (F). Cells were harvested with Accutase, sorted for CD31, detergent lysed and immunoblotted as indicated (E). Statistics: B and F, two-sided exact Wilcoxon rank-sum tests.

We therefore determined whether BMX was necessary for p130CAS activation in ECs co-seeded with CSCs, by downregulating BMX in ECs that were then co-seeded with CSCs on laminin in NBM with bFGF (3 h). The downregulation of BMX significantly inhibited p130CAS phosphorylation in ECs co-seeded with CSCs but had no effect on p130CAS phosphorylation in ECs seeded alone in CM/EC+CSC (Figure 6A&6B). To determine whether BMX was activated in ECs on co-seeding with CSCs, phospho-BMX was detected by immunofluorescence and fluorescent intensity quantitated as described for phospho-p130CAS. We found a significant increase (~3-fold) in phospho-BMX in ECs co-seeded with CSCs as compared to ECs seeded alone in CM/EC+CSC (Figure 6C&6F) although there was no significant difference in total BMX in ECs in the two conditions after Accutase harvest, CD31 sorting, lysis and blotting for BMX (Figure 6E). To further determine whether the increase in phospho-BMX was dependent on integrin αvβ3 in the ECs co-seeded with CSCs, we downregulated the β3 subunit in ECs. There was a significant inhibition of BMX phosphorylation in the ECs in which the β3 subunit was downregulated when they were co-seeded with CSCs, but the BMX phosphorylation in these ECs was unaffected when they were seeded alone in CM/EC+CSC (Figure 6C, 6D&6F). Taken together, these data indicate that BMX is necessary for the increased p130CAS activation and EC migration towards bFGF in ECs co-seeded with CSCs on laminin, but is not necessary for p130CAS activation and EC migration when ECs are seeded alone.

### RGD-peptide treatment reduces the proximity of Sox2-positive tumor cells to ECs and decreases phosphorylation of BMX and p130CAS in ECs in a xenograft model of GBM

**Figure 7:**
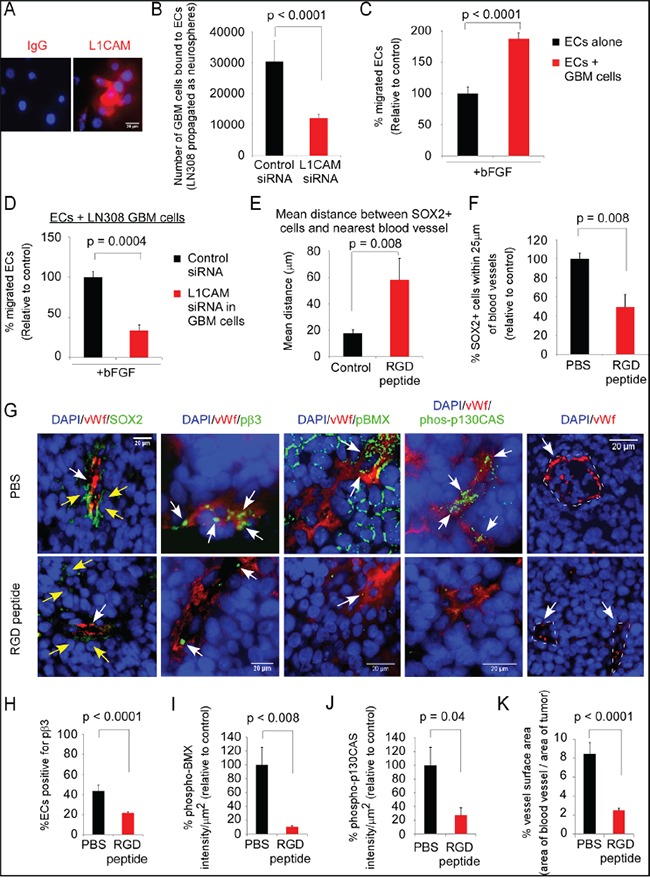
Administration of the RGD-peptide significantly reduces the proximity of Sox2-positive tumor cells to ECs, and BMX and p130CAS activation in ECs in an intracerebral xenograft model of GBM **A-D.** LN-308 human GBM cells propagated in complete NBM in suspension were fluorescent-labeled for L1CAM (Alexa-594-red) (A); allowed to adhere to a monolayer of ECs after downregulation of L1CAM (B); mixed with ECs, plated on laminin-coated filters and allowed to migrate to bFGF as in Figure 5 (C), or L1CAM was downregulated and the migration assay repeated (D). **E-K.** LN-308 cells (100,000) were injected into the nude mouse brain, tumor allowed to establish for 55 days, and cyclic-RGD-peptide (90 mg/kg) or PBS were administered for 5 days (n=5 mice/group), followed by euthanasia at 2 h post-treatment and brain harvest. Sections double-labeled for vWf (Alexa-594, red) and either Sox2, pβ3-(Y759), phospho-BMX, or phospho-p130CAS (Y234) (Alexa-488, green), and DAPI nuclear staining (G). The mean Sox2-positive tumor cell distance from vWf-positive ECs (E), percent Sox2-positive tumor cells within 25-μm of a vWf-positive vessel (F), percent ECs positive for pβ3 (H), intensity of phospho-BMX in tumor-associated ECs (I), intensity of phospho-p130CAS in tumor-associated ECs (J), and vessel surface area in the tumor (K) from RGD-peptide-treated mouse tumors relative to vehicle-treated control tumors. The intensity of phospho-BMX and phospho-p130CAS was calculated using FIJI (ImageJ) as in Figure 2 E&2F for vWf-positive blood vessels, and traced along their outer edge for each field taken at 40X magnification. Signal intensity was normalized by dividing the sum of the mean gray level by the area of the ECs in each blood vessel. Statistics: B-F, two-sided exact Wilcoxon rank-sum tests; and H-K, linear mixed regression model. Data graphed as the mean±SEM.

Others have reported that cyclic-RGD-peptide (Cilengitide) treatment prolongs survival of *scid* mice bearing intracerebral GBM tumors, and of nude rats bearing intracerebral GBM tumors when administered with radiation [37, 38]. As we had found that an RGD-peptide blocks the interaction of integrin αvβ3 on ECs with L1CAM on CSCs, we examined the effects of administration of the cyclic-RGD-peptide in an established orthotopic mouse model of GBM on the distance of Sox2-positive tumor cells from ECs and integrin αvβ3-mediated signaling in the ECs. LN-308 GBM cells were utilized as they express L1CAM when propagated as neurospheres in NBM (Figure 7A), bind to ECs in the cell-cell adhesion assay in an L1CAM-dependent manner (Figure 7B), and promote EC migration towards bFGF in an L1CAM-dependent manner (Figure 7C&7D). LN-308 cells were injected into the nude mouse brain and at day 55 administration of cyclic RGD-peptide was initiated, followed by euthanasia and brain harvest on day 60. We found a significant increase in the mean distance of Sox2-positive cells from ECs (Figure 7E&7F), and a significant decrease in the number of Sox2-positive cells within 25-μm of blood vessels (Figure 7F&7G). Also, we found significant decreases in the percent of ECs expressing pβ3-(Y759), in the intensity of phospho-BMX (BMX activation) and phospho-p130CAS in ECs, and in vessel surface area in the tumors of RGD-peptide treated mice as compared to controls (Figure 7F, 7H-7K). Phospho-BMX staining of ECs in the xenograft tumors was detected in a population of vessels, consistent with BMX expression in vessels of arterial origin [39, 40]. These data suggest that ECs interact with tumor cells in the perivascular niche through an RGD-peptide-binding integrin and that this interaction promotes BMX and p130CAS activation, thereby enhancing angiogenesis.

## DISCUSSION

Here, we describe for the first time a direct interaction of ECs with CSCs from GBM that is mediated through binding of integrin αvβ3 on ECs to the RGD-peptide in the extracellular domain of L1CAM on CSCs. This activates integrin αvβ3 on ECs and, in the presence of bFGF, results in significantly increased activation of BMX, FAK and p130CAS, increased activation of the downstream effectors ERK and JNK, and marked changes in the phenotype of the ECs. Importantly, this level of upstream and downstream effector activation was not achieved by EC seeding in CM/EC+CSC with bFGF, indicating that it cannot be attributed to a secreted factor(s). Furthermore, the increase in ERK and JNK activation was RGD-peptide-dependent suggesting a requirement for the direct interaction of ECs with CSCs rather than an effect due to integrin α6-mediated adhesion to laminin (which is RGD-peptide-independent) [reviewed in [8, 9]]. Minimal p130CAS activation was detected in CSCs, suggesting activation of another p130CAS family member, such as NEDD9(HEF1) [12, 41]. In ECs co-seeded with CSCs, we found that integrin αvβ3 is necessary for BMX activation and that BMX is necessary for p130CAS activation whereas in ECs seeded in CM/EC+CSC, αvβ3 is not necessary for BMX activation and BMX is not necessary for p130CAS activation. BMX, a cytoplasmic non-receptor tyrosine kinase, can activate signaling pathways that promote migration and angiogenesis as well as other signaling pathways [39, 40, 42–46]; thus, the increased BMX activity downstream of activated integrin αvβ3 in the ECs co-seeded with CSCs could serve to amplify and diversify signaling.

The alterations in the actin cytoskeleton that occurred in ECs that contact CSCs suggest a pro-migratory phenotype, and co-seeding ECs with CSCs on laminin significantly increased EC migration towards bFGF as compared to that observed when the ECs were seeded on laminin in CM/EC+CSC. This increased migration was dependent on integrin αvβ3 on ECs and L1CAM on CSCs, supporting our hypothesis that the direct cell-cell contact promotes EC migration towards bFGF. The possibility that the migration was mediated by integrin αvβ3 binding to the laminin substrate was ruled out by the failure of downregulation of β3 to significantly affect migration of the ECs seeded alone on laminin.

The current studies focus specifically on the effects of EC-CSC contact mediated by integrin αvβ3 on EC signaling and migration. To characterize this response, we focused on the 7-24 h time frame when cell-cell contact and directed migration were observed. We found distinct differences between the signals associated with migration of ECs induced by the direct interaction with CSCs and the signals induced by the soluble factors present in CM/EC+CSC. These differences included the requirement for BMX for cell-contact induced signaling of migration, as evidenced by the downregulation of BMX and the use of a BMX inhibitor. These differences occurred in the context of some signals that were induced in both ECs that interacted directly CSCs and by soluble factors present in CM/EC+CSC. Such shared signaling may form the framework for the migratory behavior of the ECs whether or not they come into direct contact with the CSCs and will provide a basis for further investigation of, for example, the EC migratory polarity observed without CSCs, which most likely can be attributed to the chemotactic gradient of bFGF, and the induction of unpolarized migration in the 7-24 h time frame of ECs that did not come into contact with CSCs.

Our *in vivo* animal studies indicate that treatment of an established GBM xenograft tumor with cyclic-RGD-peptide significantly increased the mean distance of Sox2-positive tumor cells from ECs as compared to controls. We also observed significant decreases in the percent of ECs with integrin αvβ3 activation, in BMX activation and p130CAS phosphorylation in ECs, and in vessel surface area in tumors from RGD-peptide-treated mice. A clinical trial of the cyclic-RGD-peptide (Cilengitide) in combination with standard chemo-radiation for patients with newly diagnosed GBM showed improvement of survival in a subset of patients [47]. Our current data indicate previously unrecognized mechanisms of action that may affect therapeutic responsiveness.

Prior studies that have shown that integrin αvβ3 cooperation with bFGF promotes angiogenesis [9, 48–50] utilized models that do not take into account the effects of direct contact with perivascular CSCs or tumor cells on integrin αvβ3 signaling in the ECs. The current studies provide insights and also raise questions that are relevant to the ongoing development of strategies for utilization of targeted therapies, and most especially those directed to integrin αvβ3, L1CAM and ERK. In terms of regulation of signaling, for example, integrin αvβ3 cooperation with bFGF in confluent monolayers of ECs has been shown to be due to a complex formed between integrin αvβ3, the tetraspanin CD9, and the junctional adhesion molecule-A (JAMA) that upon bFGF stimulation releases JAMA thereby regulating ERK activation and migration [51] but it is not known whether a similar complex is formed in the context of EC-CSC contact. Others have reported that cell surface expression of L1CAM is regulated by endocytosis, through a process that requires Numb, a negative regulator of Notch signaling [52]. In some cancers (*e.g.*, lung and breast cancer) activated ERK signaling drives alternative splicing of Numb resulting in expression of the Numb-E9 isoform that can enhance Notch signaling [53]. Also, activation of Notch signaling by its ligand Delta-like 4 promotes angiogenesis in tumors [54]. These data raise the intriguing question as to whether similar mechanisms affect cell surface L1CAM expression in GBM, and the intersection between Notch and ERK signaling and the cell interaction model that we propose in shaping tumor-associated angiogenesis. Although others have reported that, during metastasis, melanoma cells undergo transendothelial cell migration through a mechanism involving an interaction between integrin αvβ3 on melanoma cells and L1CAM on ECs [55], it should be noted that GBM tumors rarely metastasize outside of the brain [2]. Finally, it is not known if the CSC-EC interaction also promotes survival and, if so, whether it does so through a mechanism akin to that observed on binding to an extracellular substrate [56, 57].

In summary, we have identified a previously unrecognized integrin αvβ3/bFGF initiated signaling event in the perivascular niche in GBM, involving EC contact with CSCs or tumor cells that requires BMX for activation of p130CAS and promotes chemotactic migration of ECs and thereby angiogenesis.

## MATERIALS AND METHODS

### Cells

Primary human normal brain ECs were purchased (Cell Systems, Kirkland, WA), cultured in EGM medium with growth factors (SingleQuots; Lonza, Basal, Switzerland), used in the first eight passages and von Willebrand factor (vWf) expression verified repeatedly, as described [30]. Primary human CSCs or NSTCs (08387) from four different GBM xenografts (08387, 3691, 3832, 4302) were isolated and propagated as neurospheres in complete neural basal medium (NBM) (Life Technologies, Waltham, MA) without FBS, as described [58]. Re-authenticated LN-308 cells were received from Dr. Nicolas de Tribolet (Lausanne, Switzerland). Re-authentication was performed by DNA profiling of 8 highly polymorphic regions of Short Tandem Repeats using Nonaplex PCR at the Leibniz Institute DSMZ – German Collection of Microorganisms and Cell Cultures before use in these experiments. ECs were labeled with PKH26-red-fluorescent cell linker (Sigma, St. Louis, MO). Human astrocytes were purchased (Cell Systems) and cultured in DMEM with 10% FBS. U-118 MG GBM cells expressing L1LE or vector control (K1879) have been described previously [28]. The U-118/L1LE and U-118/K1879 vector control cell lines were authenticated to the HTB-15 (U-118 MG) human ATCC cell line by DNA profiling of 17 short tandem repeat (STR) loci plus the gender determining loci at the ATCC.

### Inhibitors and downregulation studies

Peptides (Peptides International, Louisville, KY) and PP2 (EMD Millipore) were purchased. Cyclic-RGD-peptide (Cilengitide) used in the mouse model was provided by Merck (Darmstadt, Germany). The FAK inhibitor PF573228 was purchased from Santa Cruz, and the BMX-Inhibitor-1 was purchased from EMD Millipore. Both were used at a final concentration of 1μM.

Small interfering ON-TARGETplus SMART pool RNA (siRNA) oligonucleotides were purchased (Dharmacon, Lafayette, CO). Pre-designed single target siRNA oligonucleotides for integrin β3 were purchased from Sigma (SASI_Hs01_00174222) and Life Technologies (s7582). Pre-designed single target siRNA oligonucleotides for L1CAM were purchased from Life Technologies (s8036, s8037, s8038). Pre-designed single target siRNA oligonucleotides for p130CAS were purchased from Life Technologies (s225161, s18373). Cells were transfected with oligonucleotides (HiPerfect, Qiagen, Hilden, Germany) and downregulation confirmed by immunoblotting [30, 59].

### Antibodies

Antibodies were purchased: monoclonal antibodies, anti-integrin αvβ3, anti-integrin β3(CD61), and anti-integrin α5β1 (Millipore, Billerica, MA), anti-human CD171 (L1CAM; BD Pharmingen, San Diego, CA), anti-integrin αvβ5 (R&D Systems, Minneapolis, MN), anti-actin, anti-β-tubulin, anti-phospho-ERK, and anti-phospho-JNK (Santa Cruz, Dallas, TX), and anti-L1CAM UJ127 (GeneTex, Irvine, CA); rabbit antibodies, anti-integrin β3 (Epitomics, Burlingame, CA), and anti-phospho-FAK (Y397), anti-total FAK, anti-phospho-BMX/Etk (Y40), anti-total BMX/Etk, and anti-total p130CAS (E1L9H) (Cell Signaling, Beverly, MA). Rabbit affinity purified phospho-specific anti-p130CAS (Y234) was generated using the following peptide: -AQPEQDE[pY]DIPRHL, corresponding to residues 227-240 in human p130CAS, by 21st Century Biochemicals, Inc (Marlboro, MA) (SFigure 4).

### Immunofluorescence

Cells were multi-labeled for immunofluorescence as described [30]. Confocal images were taken at 0.5μm steps (40X objective lens) using a Leica-TCS-SP5II-AOBS confocal microscope (NDD detector).

### Cell-cell adhesion

Adhesion assay buffer: (140 mM NaCl, 5.4 mM KCl, 5.56 mM D-glucose, 10 mM Hepes, pH 7.4, 1mM MgCl_2_, 100uM MnCl_2_, 1% BSA). Before cell seeding, the plate was blocked with 5% BSA (30 min). ECs were seeded overnight on collagen type I (MP Biomedicals) and the EC monolayer was confluent prior to beginning the assay. CSCs remaining attached to ECs were calculated based on a CSC standard curve (SFigure 1A).

### Matrigel^®^ EC network formation

Cells were seeded onto polymerized Matrigel^®^ in complete NBM. Pictures (5X-magnification) from three independent assays were taken using a Zeiss A1 microscope. CM from CSCs was prepared by seeding CSCs on laminin in complete NBM (72 h).

### Motility assays

For the 2D assay, cell culture inserts and the Chemotaxis and Migration Tool 2.0 (Ibidi, Madison, WI) were used. Inserts were coated with 20 μg/ml laminin (R&D Systems). PKH26-red-fluorescent-ECs in EGM media were plated on one side of the insert, and GFP-CSCs in complete NBM on the other side. Subsequently, the media was replaced with complete NBM, the insert removed, and live-imaging of cells into the gap initiated using a 10X objective lens and a motorized-stage (Leica-DMI6000-ImageEM/Orca-R2-ImageProPlus). Manual Tracking (Image J) was used to trace EC paths into the 500 μm gap [60]. ECs trajectories were projected onto the XY plane and the average velocity calculated for individual and total trajectories [60].

For analysis of CSCs/ECs migrating into the wound gap, an automated algorithm was generated by ImageIQ (Cleveland, OH) designed to batch process and analyze time-lapse, multi-channel stacks within ImagePro-Plus-7.0.

For the Transwell^®^ assay, cells were seeded on the upper filter surface (Transwell chambers, 3-μm pore, Corning), allowed to migrate, cells on the upper surface removed, and cells on the lower filter surface washed, fixed [11], photographed and counted in 5-10 fields with a 20X objective (Leica-DFC425C-QImaging-Q15729-QCapturePro).

### qRT-PCR

RNA was harvested, reverse transcribed, and quantitative PCR performed using the Syber Green system; levels of mRNA were normalized to GAPDH, as described [30].

### Animal studies

Animal experiments were carried out as described [61] with the approval of the Institutional Animal Care and Use Committee at the University Hospital Zurich (Zurich, Switzerland). LN-308 cells (10^5^ cells in 2-μl) were stereotactically injected into the striatum of 6-12 week-old anesthetized athymic CD1 nude mice (Charles Rivers Laboratories, Wilmington, MA).

### Statistics

All statistical analyses were performed/overseen by the Biostatistician (ASN). The statistical test used is stated in the figure legend; a *p* value <0.05 was considered significant.

## SUPPLEMENTARY FIGURES


