# Supplementary material for: Direct contact with perivascular tumor cells enhances integrin αvβ3 signaling and migration of endothelial cells
Source: Oncotarget. 2016 May 30;7(28):43852–67. doi: 10.18632/oncotarget.9700 (PMC5190064; doi:10.18632/oncotarget.9700)
Supplement: Supplementary file 1 [file oncotarget-07-43852-s001.pdf]

Direct contact with perivascular tumor cells enhances integrin αvβ3 signaling and migration of endothelial cells

SUPPLEMENTARY FIGURES

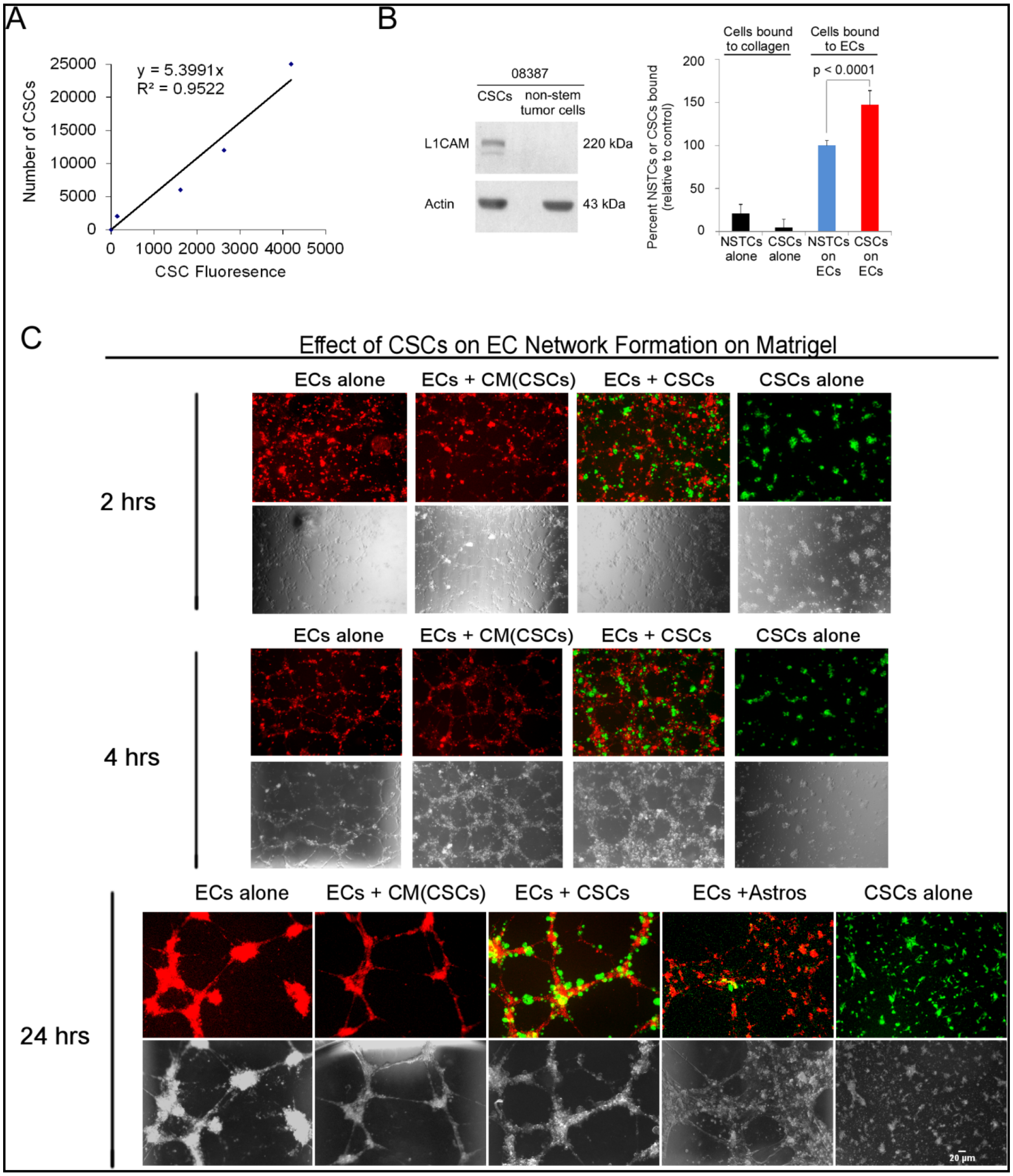

Supplementary Figure S1: Controls for the cell-cell adhesion assay; enhanced binding of CSCs to ECs compared to paired NSTCs; and an association of ECs and CSCs after co-seeding on Matrigel. A. Representative standard curve of the fluorescence emitted from increasing numbers of adherent GFP-CSCs in the cell-cell adhesion assay. **B**. Immunoblotting of adherent CSCs and paired NSTCs (08387) for L1CAM; Unlabeled-ECs (5x10^4/well) were seeded in serum-free adhesion assay buffer on plates coated with 20 µg/mL collagen, allowed to attach overnight (replicates of five). GFP-CSCs-(08387) or paired NSTC (08387) (3x10^4) were plated over the ECs, allowed to adhere (30 min), washed 3X with PBS and fluorescence detected using a fluorometer (485nm absorption, 535 nm emission). **C**. Red-fluorescent ECs were seeded on top of Matrigel in complete NBM (ECs alone), in CSC conditioned-media (CM), or when mixed with CSCs. An association between ECs and CSCs is visualized at 2, 4 and 24 h. (5X objective lens).

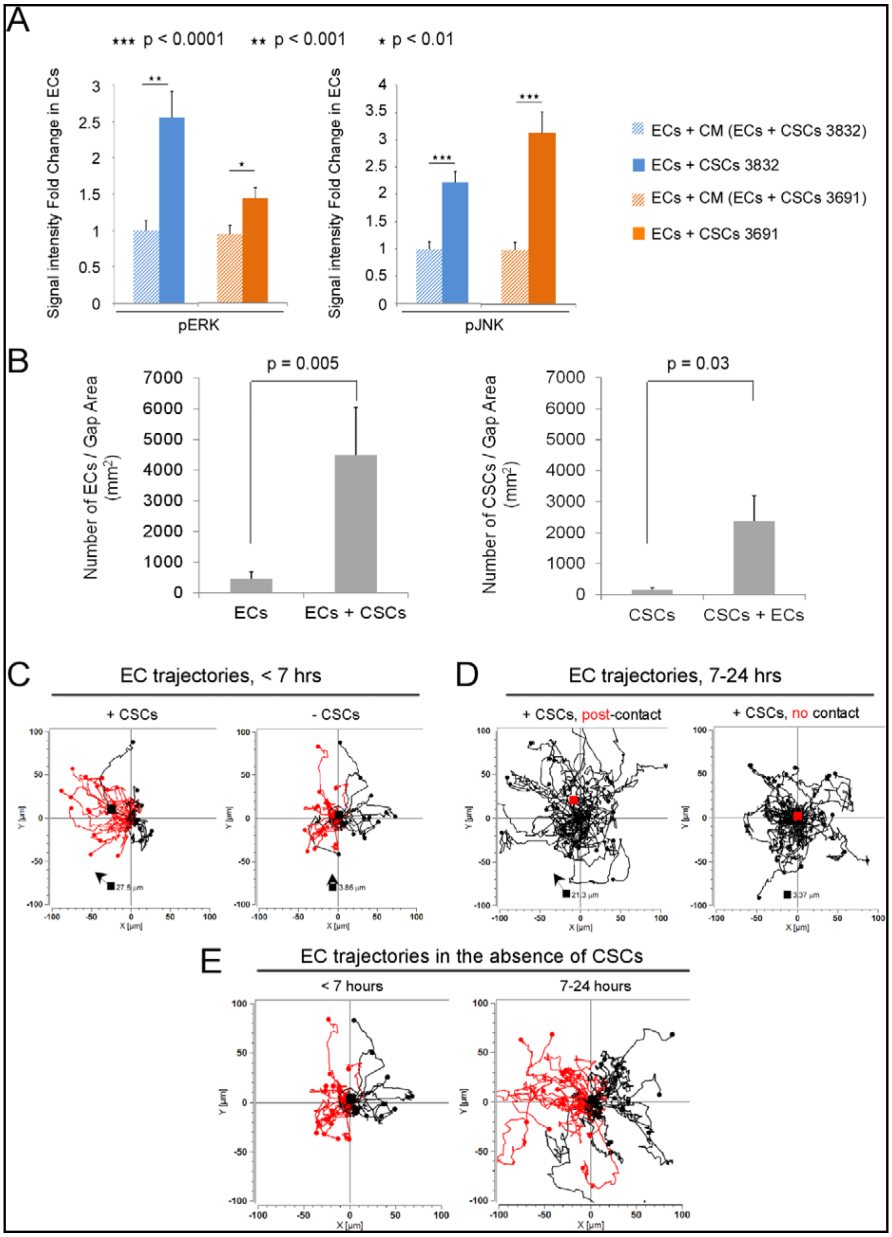

Supplementary Figure S2: Co-seeding of ECs and CSCs significantly increases the activation of ERK and JNK in ECs; and CSCs increase the directional 2D motility of ECs through secreted factors and through direct contact. A. ECs in CM/EC+CSC or mixed directly with CSCs (3832 or 3691) were seeded onto laminin in NBM with bFGF (10 ng/ml) and no EGF (3 h), double-labeled for pERK or pJNK (Alexa-594, red) and for vWf (an EC marker, Alexa-488, green) by double-label immunofluorescence, followed by the calculation of signal intensity using FIJI (ImageJ) based on the sum of the mean gray level (intensity) output of all pixels of the area within the enclosed cell traced along its outer edge. Intensity was normalized by dividing the sum of the mean gray level by the area of the cell. Data are graphed as the mean±SEM. Statistics: two-sided Wilcoxon rank-sum tests, n=50. **B.** Red-fluorescent ECs and GFP-CSCs were seeded on either side of an 500 µm insert onto laminin, at 18 h the insert removed, and live-imaging of migration into the 500 µm gap performed q 15 min (24 h). The effect on EC and CSC migration into the gap when plated opposite of each other is graphed as the mean±SEM number of cells/mm^2 in the gap. Statistics: exact two-sided Wilcoxon rank-sum tests. **C.** Tracks (trajectories) of ECs (n=30) with and without the presence of CSCs in the first 7 h of migration before possible cell-cell contact. **D.** The trajectories and displacement of ECs plated opposite of CSCs between 7-24 h after contact with CSCs (left panel) or in the absence of CSC contact (right panel) during the same time frame. **E**. Trajectories of the random migration pattern of ECs throughout the entire 24 h in the absence of CSCs.

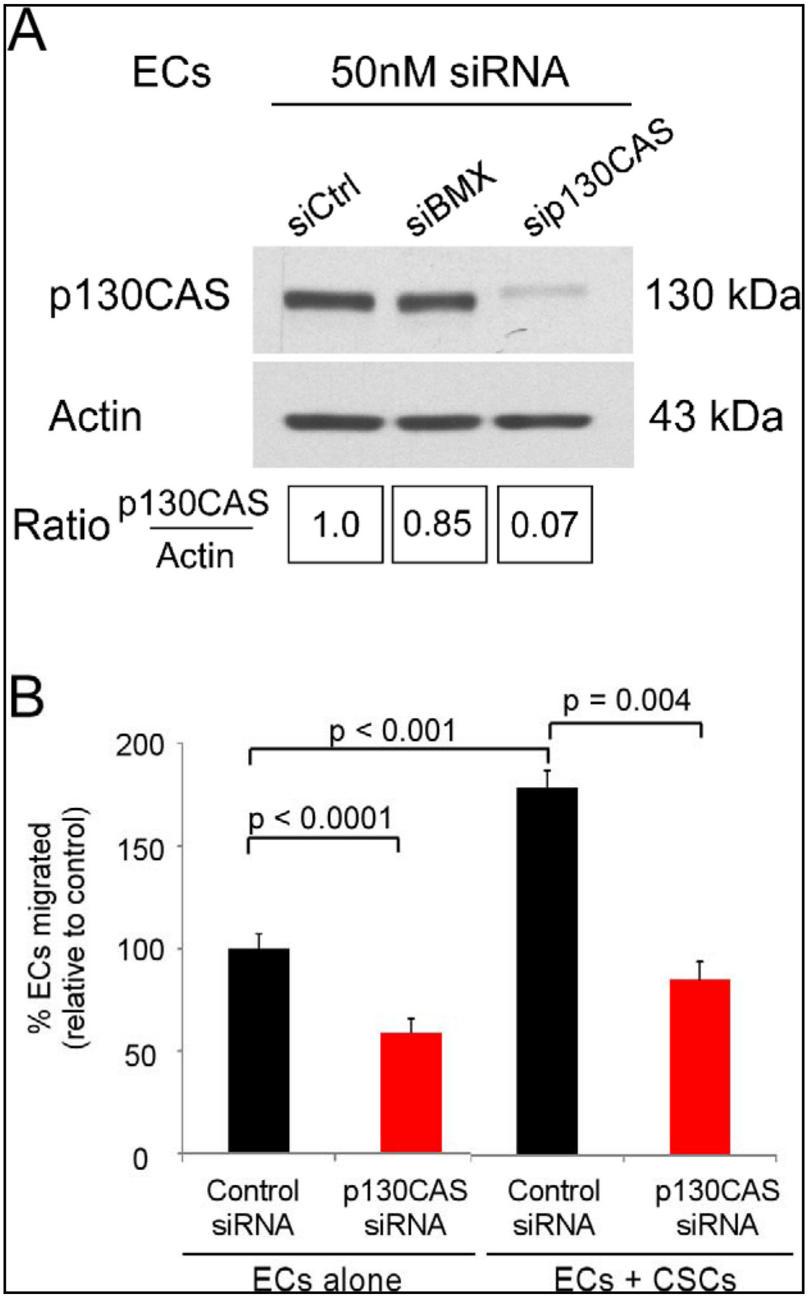

Supplementary Figure S3: The direct interaction of ECs and CSCs promotes EC migration towards bFGF and requires p130CAS. A. ECs were treated with p130CAS and BMX pooled siRNA or with control siRNA for 48 h detergent lysed and immunoblotted for the indicated antibodies. **B.** Filters (3-µm pore) were coated on both sides with 20 µg/mL laminin. Red fluorescent- ECs ($3x10^4$) were seeded alone or mixed with GFP-CSCs-(08387) ($3x10^4$) and co-seeded in growth factor-free NBM with 1% BSA on top of the filter. Growth factor-free NBM with 10 ng/ml bFGF was placed in the bottom chamber, and the cells allowed to migrate (37oC, 5% CO2). At 6 h, cells were removed from the upper filter surface and cells on the lower filter surface were washed, fixed, photographed and counted. ECs were treated with the indicated siRNA or control siRNA, washed and seeded alone or with GFP-CSCs.

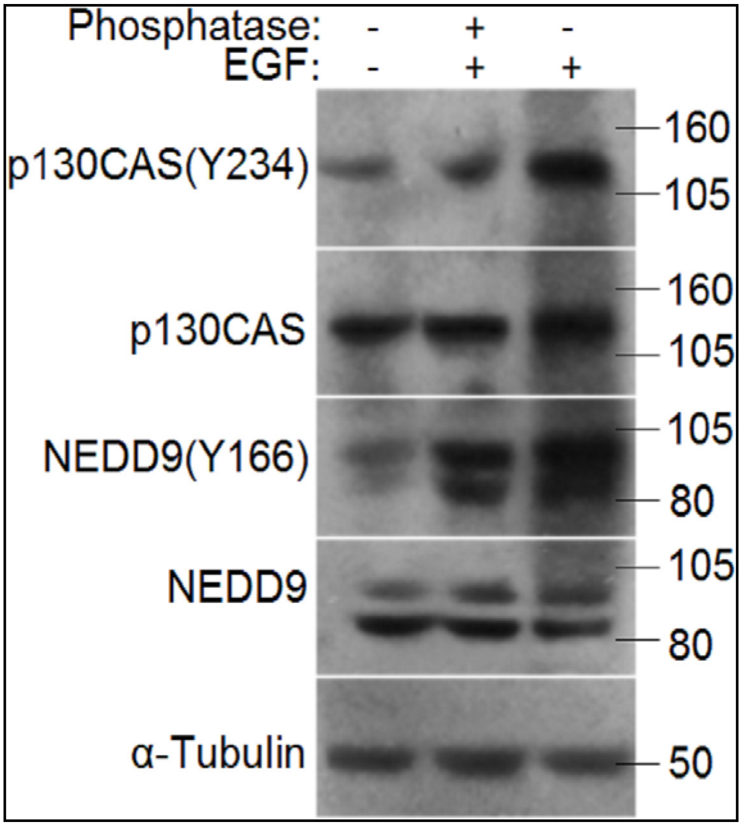

Supplementary Figure S4: Specificity of rabbit affinity purified anti-phospho-p130CAS (Y234) antibody. Rabbit phospho-specific antibody towards p130CAS (Y234) was generated using the following peptide: -AQPEQDE[pY]DIPRHL, corresponding to amino acids 227-240 in human p130CAS protein, and affinity purified as described (1, 2). The antibody was tested for specificity using MCF7 cells that were serum starved for 24h, treated with EGF for 30 min, lysed and subjected to disulfide-reduced SDS-PAGE (1, 2). Lysate was also treated with a cocktail of recombinant phosphatases (PP1, PP2A, AP, SHIP2) for 5 min to dephosphorylate proteins before addition of the gel loading buffer (1, 2). The anti-phospho-p130CAS (Y234) antibody (dilution 1:1000) detects a band migrating at 130-kDa and does not cross react with phosphorylated NEDD9 (HEF1) protein that migrates as a doublet between 80 and 100-kDa. The mouse mAb used for detection of total p130CAS was purchased (BD Biosciences, 1:5000 dilution) and directed towards the C-terminus. Rabbit affinity-purified anti-phospho-NEDD9 (Y166) antibody (dilution 1:500) and mouse mAb 2G9 anti-NEDD9 (1:5000 dilution) used to detect phosphorylated and total NEDD9 protein, respectively, have been described previously (1, 2).

1. Pugacheva EN, Golemis EA. The focal adhesion scaffolding protein HEF1 regulates activation of the Aurora-A and Nek2 kinases at the centrosome. Nat.Cell Biol. 2005;7:937-46.
2. Kozyreva VK, McLaughlin SL, Livengood RH, Calkins RA, Kelley LC, Rajulapati A, Ice RJ, Smolkin MB, Weed SA and Pugacheva EN. NEDD9 Regulates Actin Dynamics through Cortactin Deacetylation in an AURKA/HDAC6-dependent Manner. Mol Cancer Res. 2014;12:681-93.
